# An Intense and Short-Lasting Burst of Neutrophil Activation Differentiates Early Acute Myocardial Infarction from Systemic Inflammatory Syndromes

**DOI:** 10.1371/journal.pone.0039484

**Published:** 2012-06-25

**Authors:** Norma Maugeri, Patrizia Rovere-Querini, Virgilio Evangelista, Cosmo Godino, Monica Demetrio, Mattia Baldini, Filippo Figini, Giovanni Coppi, Massimo Slavich, Marina Camera, Antonio Bartorelli, Giancarlo Marenzi, Lara Campana, Elena Baldissera, Maria Grazia Sabbadini, Domenico Cianflone, Elena Tremoli, Armando D’Angelo, Angelo A. Manfredi, Attilio Maseri

**Affiliations:** 1 Università Vita-Salute San Raffaele and San Raffaele Scientific Institute, Milano, Italy; 2 Istituto Consorzio Mario Negri Sud, Santa Maria Imbaro, Italy; 3 Centro Cardiologico Monzino, Milano Italy; 4 Heart Care Foundation, Florence, Italy; Albert Einstein College of Medicine, United States of America

## Abstract

**Background:**

Neutrophils are involved in thrombus formation. We investigated whether specific features of neutrophil activation characterize patients with acute coronary syndromes (ACS) compared to stable angina and to systemic inflammatory diseases.

**Methods and Findings:**

The myeloperoxidase (MPO) content of circulating neutrophils was determined by flow cytometry in 330 subjects: 69 consecutive patients with acute coronary syndromes (ACS), 69 with chronic stable angina (CSA), 50 with inflammation due to either non-infectious (acute bone fracture), infectious (sepsis) or autoimmune diseases (small and large vessel systemic vasculitis, rheumatoid arthritis). Four patients have also been studied before and after sterile acute injury of the myocardium (septal alcoholization). One hundred thirty-eight healthy donors were studied in parallel. Neutrophils with normal MPO content were 96% in controls, >92% in patients undergoing septal alcoholization, 91% in CSA patients, but only 35 and 30% in unstable angina and AMI (STEMI and NSTEMI) patients, compared to 80%, 75% and 2% of patients with giant cell arteritis, acute bone fracture and severe sepsis. In addition, in 32**/**33 STEMI and 9**/**21 NSTEMI patients respectively, 20% and 12% of neutrophils had complete MPO depletion during the first 4 hours after the onset of symptoms, a feature not observed in any other group of patients. MPO depletion was associated with platelet activation, indicated by P-selectin expression, activation and transactivation of leukocyte β2-integrins and formation of platelet neutrophil and -monocyte aggregates. The injection of activated platelets in mice produced transient, P-selectin dependent, complete MPO depletion in about 50% of neutrophils.

**Conclusions:**

ACS are characterized by intense neutrophil activation, like other systemic inflammatory syndromes. In the very early phase of acute myocardial infarction only a subpopulation of neutrophils is massively activated, possibly via platelet-P selectin interactions. This paroxysmal activation could contribute to occlusive thrombosis.

## Introduction

Platelet and endothelial activation, associated with plaque instability, are important mechanisms in the pathogenesis of acute coronary syndromes (ACS) [1]–[5]. Activated platelets and endothelium interact with circulating leukocytes [6]–[16], which are also activated in ACS generating a positive feed-back loop.

Activated platelets and neutrophils appear in the peripheral circulation after the onset of acute symptoms of infarction, but gradually disappear during the subsequent hours [1], [10], [11], [17] at variance with platelet-monocyte heterotypic aggregates, which persist longer [1], [17]. Neutrophils also represent the major cellular component of coronary thrombi [2], [4], [13], [18], [19] and their activation is associated with massive myeloperoxidase (MPO) release [3], [10], [11], [20] [21]–[22]. In turn the peak concentration of circulating MPO is reached in the very early hours after infarction [3] and MPO is found in unstable coronary plaques at post mortem [2], [4], [13], [19].

Given the short life of circulating neutrophils the release of their MPO content can only reflect the effects of stimuli active during the previous 6–8 hours of their life time. Thus, measurement of neutrophil MPO content at different times after the onset of symptoms could provide information on the time-course as well as clues on distinctive features of inflammatory stimuli associated with coronary instability. We have studied neutrophil MPO content and its relation to platelet and monocyte activation in patients with segment T elevation myocardial infarction (STEMI), with non elevation myocardial infarction (NSTEMI) and with unstable angina, sampled before any therapeutic intervention. As controls, we studied patients with chronic stable angina (CSA) before and after coronary angioplasty. In addition, for comparison, we have studied patients with well established inflammatory conditions: Giant Cell Arteritis, Polymyalgia Rheumatica, ANCA-associated small-vessel vasculitis, rheumatoid arthritis, severe sepsis, and acute traumatic bone fractures.

We have observed a large reduction of the average MPO content in most patients but not in matched healthy controls or in subject with CSA. In addition, we have identified a distinctive feature of neutrophil activation in the very early hours after acute myocardial infarction, reflecting a paroxysmal response to an intense, short lasting trigger. Platelet P-selectin expression was correlated with neutrophil activation, which in turn may contribute to thrombus formation [15], [16], [23].

## Materials and Methods

### Patients and Controls

Patients and healthy donors (330 consecutive subjects) were studied in blind. We studied 69 patients with acute coronary syndromes before any therapeutic intervention: 54 with acute myocardial infarction (AMI) (34 with STEMI and 20 with NSTEMI), and 15 patients with unstable angina, studied less than 12 hours (mean±SD 4.0 hours ±30 min) from the onset of symptoms. Unstable angina was defined as typical chest pain at rest without elevation of creatine kinase or troponin I. AMI was defined on the basis of chest pain at rest, ECG recording and elevation of creatine kinase or troponin I. STEMI patients were all those that had electrocardiography evidence of at least 2 mm in at least 2 consecutive leads of ST segment elevation. From each patient, clinical data were obtained, including age, sex, hypercholesterolemia (total plasma cholesterol concentration >200 mg/dl or ongoing lipid-lowering drug therapy), hypertension (history of elevated blood pressure requiring antihypertensive therapy), diabetes mellitus (history of diabetes or fasting plasma glucose >126 or >200 mg/dl 2 h after a meal), active smoking (>5 cigarettes daily), and overweight/obesity (body mass index >25 kg/m^2^), C-reactive protein (CRP) and erythrocyte sedimentation rate (ESR). To analyze the time course of neutrophil activation in acute coronary syndromes, 36 AMI patients and 10 patients with unstable angina were again studied at 24, 36 and 48 hours after Percutaneous Coronary Intervention (PCI). We also studied 69 patients with chronic stable angina (CSA) at the time of coronary angiography: 15 of them were re-investigated 4 hours after PCI. All CSA patients had evidence of myocardial ischemia during stress tests or of angina recurrence. PCI was performed in case of: *i)* angiographic evidence of critical stenosis (>75%) or *ii)* lesions with stenosis <75% but characterized by a reduced myocardial fractional flow reserve (cut-off <0.75). Four patients with hypertrophic obstructive cardiomyopathy were studied before and four hours after absolute ethanol had been introduced into a major septal artery to create a controlled septal infarction [24]. We also studied five patients with bone fractures, seven patients with severe sepsis, 11 patients with ANCA-associated small-vessel vasculitis, eight patients with polymyalgia rheumatica, eight patients with giant cell arteritis and eleven patients with rheumatoid arthritis previous any treatment, classified according to the American Rheumatism Association 1987 revised criteria [25]. Patient clinical and demographic characteristics are summarized in **Tables 1** **,** **2** **and** **3** **.** In parallel, 138 healthy donors were also studied (**Table S1**). In all cases exclusion criteria were: age <18 years, serum creatinine >2 mg/dL, anemia and history of cancer. All patients and controls signed an informed consent form (see ethics statement).

**Table 1:** Characteristics of patients with coronary artery diseases.

	UA*n* 15	AMI*n* 54	CSA*n* 69
Age, median (range)	61.5 (48–70)	61.7 (42–71)	60.1 (45–72)
Sex: M/F	12/3	47/7	44/15
CRP (mg/L)	4.0±0.3	4.8±0.8	1.2±0.3
ESR (mm/h)	2.1±0.4	15.8±1.6	2.3±0.3
Initial troponin I	0.2±0.1	1.2±0.2	n.a.
Peak troponin I	n.a.	30.6±6.6	n.a.
***Risk Factors***			
Cholesterol (mg/dL)	198.1±10.3	203.0±7.11	193.0±11.7
Diabetes	2/15	4/54	11/69
Hypertension	3	24	35
Current smoking	5	24	9
Obesity	3	6	4
***N° of involved coronary arteries: % patients***			
**0**	25%	12%	0%
**1**	25%	36%	10%
**2**	25%	32%	30%
**3**	25%	20%	60%
***History***			
Previous AMI	2	0	17
Previous CABG	0	0	15
Previous PCI	0	0	23
Ejection fraction	56.7±4.1	47.9±9.3	54.4±6.8
Aspirin	15	25	69
Statins	5	19	41

Demographic and clinical data are referred to the time of blood sample collection. CABG: Coronary Artery Bypass Graft. PCI: Percutaneous Coronary Intervention.

**Table 2:** Characteristics of patients with systemic inflammatory syndromes.

	ANCA-associated small-vessel vasculitis	Giant Cell Arteritis	Polymyalgia rheumatica	Rheumatoid Arthritis
*n*	11	8	8	11
Sex M/F	9/11	2/6	2/6	4/7
Age (years)	49.5 (36–78)	71 (63–87)	70 (64–81)	59.5 (32–78)
Disease duration (months)	108±11.1	10.5±2.2	29.4±6.2	23.9±8.9
CRP (mg/L)	17.8±5.5	11.5±2.5	13.3±3.1	32.2±13.6
ESR (mm/h)	37.1±11.3	32.8±4.1	40.4±8.2	44.6±11.4
Disease activity (active n, %) ¶	8 (72.7%)	6 (75%)	5 (62.5%)	8 (72.7%)
Prednisone (mg/day)	19.1±5.1	12.9±2.5	4.1±1.8	1.3±0.7
Immnosuppressive drugs (n)	Cyclophosphamide (1)Rituximab (1)	Azathioprine (2)	Methotrexate (1)	–
Organ involvement (n) **	ENT (11)1ung nodules (5)PNS (5)Nephritis (4)Arthritis (4)Cranial nerve neuritis (1)Orchitis (1)	Headache (4)Myalgias (1)Aortitis (1)Past optic nerve ischemia (2)	Myalgias (8)	11 Arthritis (11)Bone erosions (5)Lung nodules (1)

Demographic and clinical data are referred to the time of blood sample collection. If not otherwpone.0039484.g004.tifise specified, means±SEM are shown.

** number of patients with disease-related organ involvement. ENT  =  ear, nose, throat; PNS  =  peripheral nervous system.

¶ number (%) of patients with active disease (as assessed by the Rheumatologist on the basis of symptoms, inflammatory markers and, when available for the specific disease, disease activity scores).

**Table 3:** Characteristics of septic patients.

Patient/sex/age(years)	primary infection	cultures (microorganism/material)	Shock(lactate) **	OrganInvolvement (present)^ ¶^	CRP (mg/l)	organinvolvement(following)^¶^	outcome ***
1/F/60	pneumonia	S. aureus/blood	+ (3.52)	Res, Ren, Hep	277	(DIC)	A
2/F/69	diverticulitis	Staphylococcus spp/blood	–	–	473	–	A
3/F/86	colitis	C. difficile/fecies	–	–	32.9	–	A
4/M/63	bilateral pneumonia	S. marcescens/bronchoalveolar fluid	+ (2.57)	Ren	204	Hep, DIC	D
5/F/81	pneumonia	S. aureus/bronchoalveolarfluid + blood	–	Res, Ren	141	–	D
6/F/79	urinary tract infection	P.aeruginosa/blood	+ (2.90)	Ren, Hep	225	–	A
7/M/82	aspiration pneumonia	E. coli/bronchoaspirate +blood	+	Resp	220	(DIC)	D

When not otherwise specified, demographic and clinical data are referred to the time of blood sample collection, and means ± SEM values are shown for each group; + : present; − : absent;

** septic shock: when present, lactate concentration (mmol/L) is shown in brackets; ¶ involved organs at the time of blood sample collection (present) or additional organ involvement in the following 3 days (following); Res: respiratory failure; Ren: renal failure, Hep: hepatic failure; DIC  =  Disseminated Intravascular Coagulation;

*** outcome A: alive, D: dead.

### Blood Sampling and Processing

Venous blood was obtained using a 19-gauge butterfly. Blood was first drawn in a vacutainer tube to obtain serum followed by the collection of 3 mL into a tube containing sodium citrate and a cocktail of antiproteases (sodium EDTA, N-ethylmaleimide and aprotinin) used to study the neutrophil MPO content and cellular markers of activation by flow cytometry and confocal microscopy as previously described [9], [10], [11], [26]. All determinations were performed in blind for the laboratory operators.

**Table 4:** Neutrophil myeloperoxidase (MPO) content.

	n	Neutrophils count(×10^3^/µL)	Average MPOcontent(MFI)	MPO depletion(% neutrophils)	LowMPO content(% neutrophils)	NormalMPO content(% neutrophils)	Trimodal MPO content(N°/total)
Healthy volunteers	138	3,6±0,8	149.3±14.0	<1	1.2±0.9	96.4±1.4	0/138
ACS							
STEMI	33	9.2±0.6^§^	56.1±5.8^§^	21.5±5.4^§^	45.6±7.2^§^	30.6±7.1^§^	32/33
NSTEMI	21	7.9±0.5^§^	73.2±10.4^§^	11.8±4.1^§^	56.9±6.5^§^	29.9±6.2^§^	9/21
UA	15	5.2±0.5¶	91.7±12.0*	<1	60.0±6.2^§^	36.5±11.4^§^	0/15
CSA							
before PCI	69	4.0±2.8	134.1±26.9	<1	5.8±1.6*	91.2±4.7	0/54
after PCI	15	3.8±0.3	133.1±8.7	<1	11.8±4.0¶	87.5±5.7	0/15
Septal Alcholization							
before	4	3.6±0.7	131.3±11.8	<1	4.3±1.8	92.8±2.8	0/4
after	4	4.8±0.2	170.8±10.9	<1	2.5±1.3	97.2±1.8	0/4
Acute traumatic bone fracture	5	12.8±0.3^§^	116.2±12.8	<1	23.9±9.1^§^	75.2±9.3*	0/5
ANCA-associated small-vessel vasculitis	11	7.8±1.4^§^	87.9±9.9¶	<1	59.6±8.3^§^	36.5±8.4^§^	0/11
Rheumatoid Arthritis	11	7.6±0.9^§^	80.0±11.7¶	<1	74.5±9.1^§^	19.1±3.8^§^	0/11
Giant Cell Arteritis	8	5.1±0.9*	84.4±18.6¶	<1	16.0±11.3¶	80.4±8.6	0/8
Polymyalgia Rheumatica	8	5.7±1.5*	49.6±14.9^§^	<1	63.2±19.3^§^	22.3±7.1^§^	0/8
Sepsis	7	22.2±4.4^§^	61.9±8.8^§^	<1	97.0±3.5^§^	2.0±2.1^§^	0/7

MPO =  myeloperoxidase. MFI: mean fluorescence intensity revealed within neutrophils (CD45+, CD66b+, CD14− cells). ACS: acute coronary syndromes. STEMI: segment T elevated myocardial infarction. NSTEMI: no STEMI. UA: unstable angina. ANCA: anti neutrophils cytoplasmatic antigens. Statistically different from the healthy volunteers * = *P*<0.05;

¶ = *P*<0.01 and ^§^ = *P*<0.001.

**Figure 1:**
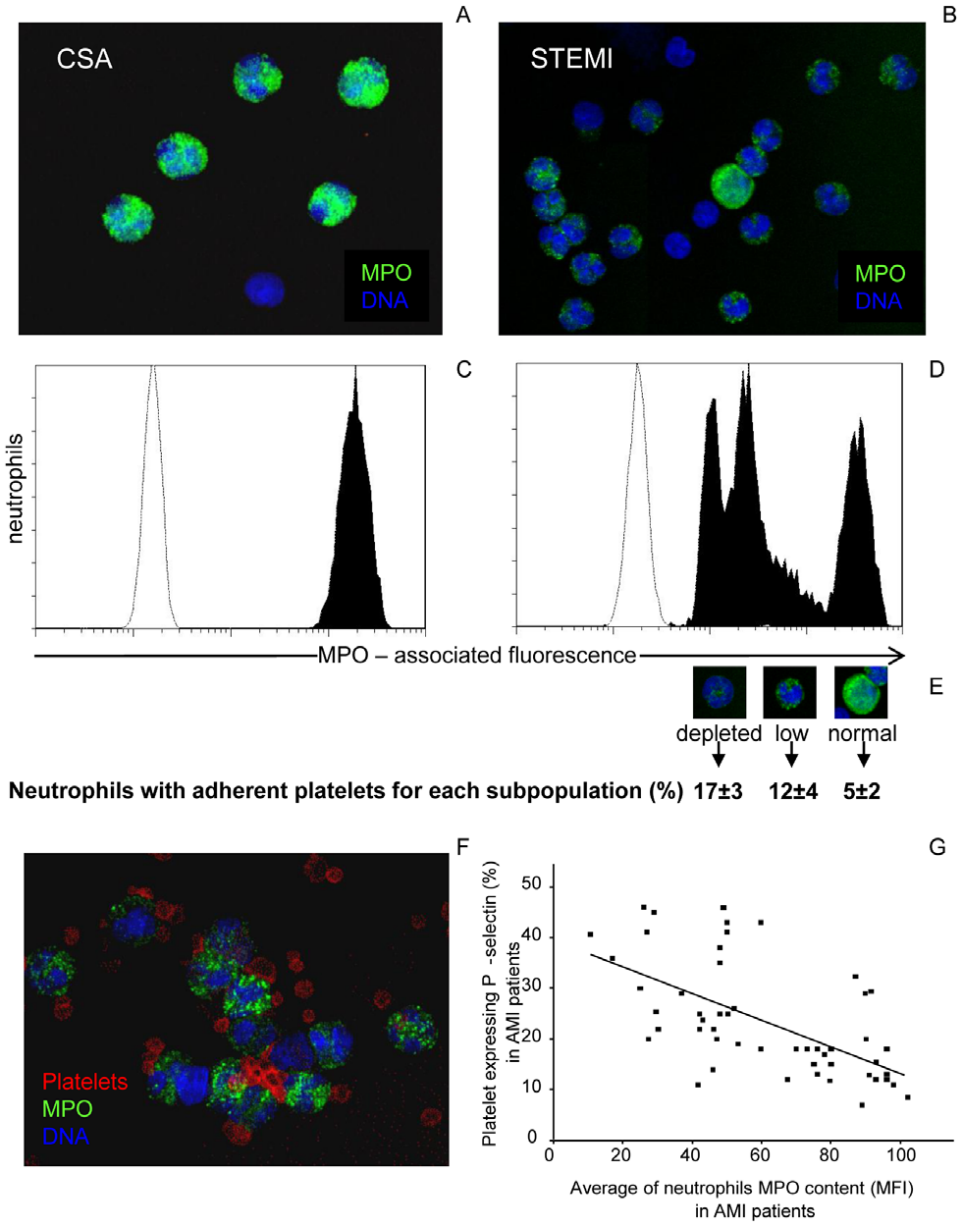
Heterogeneity of myeloperoxidase content in circulating neutrophils from patients with acute myocardial infarction is associated to platelet adhesion and activation. Intracellular MPO content in circulating neutrophils and platelet-neutrophil heterotypic aggregates were evaluated in whole blood samples by four-color flow cytometry. Venous blood samples were obtained within the first 6 hours of onset of symptoms. For confocal determinations, monoclonal antibodies against MPO were labeled with Alexa Fluor 488 (green) and against platelet glycoprotein Ib with Alexa Fluor 546 (red). Hoechst (blue) was used for counterstaining of nuclei. *Panel A.* Representative confocal images illustrate the MPO (green) content observed in circulating neutrophils of a patient with chronic stable angina. *Panel B*. Representative confocal images illustrate the heterogeneity in the MPO (green) content observed in circulating neutrophils of a patient with acute myocardial infarction. *Panel C-D.* The cytofluorimetric histogram shows the trimodal pattern MPO-associated distribution (black) in AMI but not in CSA, *vs* isotype control (white). *Panel E:* confocal images of neutrophils with complete MPO depletion, low or normal MPO content. Results are the mean value ± SEM of heterotypic aggregates observed in each neutrophil subpopulation. *Panel F*: confocal microscopy of aggregates between platelet (red) and degranulated neutrophils from a representative patient with AMI. *Panel H.* Correlation analysis indicates the association between platelet activation and neutrophil myeloperoxidase content in patients with AMI: platelet P-selectin expression (% of CD61^+^ cells, y axis) are plotted against the neutrophil intracellular MPO content (MFI-within CD66b^+^ cells, x axis; R = 0.6, *P*<0.0001).

### Reagents and Monoclonal Antibodies (mAbs*)*

Thrombofix, mAbs against human CD14 (clone RMO52), human CD42a (platelet glycoprotein Ib, clone SZ2), human CD45 (clone J33), CD61 (platelet glycoprotein IIIa, clone SZ21), human CD66b (clone 80H3), and human MPO (clone CLB-MPO-1) and the isotype-matched control mAb (clone 679.1Mc7) were from Instrumentation Laboratories (Milan, Italy). FACs Lysing Solution mAbs against human P-selectin (clone AK-4), against mouse P-selectin (clone RB40.34) against mouse CD61 (clone 2C9.G2), mouse CD14 (clone mC5-3) and mouse CD45 (clone 30-F11) and appropriate isotypic control mAbs were from Becton Dickinson (Milan, Italy). Isotype-matched control were from Beckman Coulter (Milan, Italy); mAbs against mouse neutrophils (clone 7/4), against mouse MPO, against human MRP8/14 (clone 27E10) and its isotype control were from Abcam Inc (Milan, Italy). The Zenon IgG Labeling kits were from Invitrogen, (Milan, Italy). The FIX & PERM kit was from Caltag (Turin, Italy). mAb 327c recognizes an activation-dependent epitope in the I domain of the ß2 integrin subunit [27].

**Table 5:** Markers of cellular activation.

	n	Neutrophil Mac-1 transactivation(% CD66b^+^)	Monocyte Mac-1 transactivation(% CD14^+^)	Neutrophil MRP8.14 (% CD66b^+^)	Platelet P-selectin expression (% CD61^+^)	Platelet – neutrophil aggregates (% CD66b^+^)	Platelet – monocyte aggregates (% CD14^+^)
Healthy volunteers	138	9.8±1.1	7.1±3.3	3.1±1.0	6.8±0.6	7.3±0.8	4.3±1.2
ACS							
STEMI	33	47.6±5.2^§^	56.0±11.1^§^	31.4±5.2^§^	22.6±2.1^§^	18.1±1.4^§^	16.0±1.9^§^
NSTEMI	21	38.2±9.8^§^	41.0±8.5^§^	27.9±3.1^§^	23.3±3.5^§^	15.2±1.8^§^	15.9±2.0^§^
UA	15	38.4±9.2^§^	39.1±9.1^§^	20.1±5.3^§^	14.3±2.5¶	12.2±1.6*	11.0±1.5*
CSA							
before PCI	69	9.4±1.3	6.1±0.7	4.1±1.6	7.4±0.9	6.7±0.5	4.4±0.4
after PCI	15	9.0±2.1	9.3±1.1	4.9±2.0	7.7±1.0	8.2±0.9	4.1±0.5
Septal alcoholization							
before	4	4.9±0.6	4.6±0.5	6.3±2.6	10.4±4.2	13.8±2.9	10.6±0.6
after	4	10.3±3.1	9.8±4.2	12.9±4.1	8.3±2.2	8.1±4.0	6.4±3.9
Acute traumatic bone fracture	5	10.1±3.2	5.9±1.1	2.3±1.2	6.9±3.8	6.7±4.7	4.8±3.8
ANCA-associated small-vessel vasculitis	11	44.2±5.5^§^	37.9±4.5^§^	38.2±3.2^§^	24.4±5.2¶	8.3±1.8	6.3±1.4
Rheumatoid Arthritis	11	52.1±3.7^§^	49.1±7.1^§^	21.6±6.9^§^	25.6±3.4^§^	15.7±2.6¶	8.7±2.0*
Giant Cell Arteritis	8	n.d.	n.d.	n.d	18.3±3.4*	18.1±3.9^§^	12.5±2.2¶
Polymyalgia Rheumatica	8	n.d.	n.d	n.d	9.1±3.5	13.3±2.1¶	7.4±1.5*
Sepsis	7	66.2±11.1^§^	64.3±9.6^§^	43.2±9.1^§^	14.4±2.6*	14.1±5.9*	4.4±1.1

ACS: acute coronary syndromes. STEMI: segment T elevated myocardial infarction. NSTEMI: no STEMI. UA: unstable angina. ANCA: anti neutrophils cytoplasmatic antibodies. n.d =  not determined. Statistically different from the healthy volunteers * = *P*<0.05;

¶ = *P*<0.01 and ^§^ = *P*<0.001.

### Flow Cytometry

Measurement of the neutrophil MPO content was performed as described [9], [10], [11], [26]. Briefly, whole blood samples were labeled with mAbs against CD45, CD14 and CD66b, treated with by FACs lysing solution, washed, resuspended in cold PBS, fixed and permeabilized by Fix&Perm and labeled with mAb against MPO, or the specific isotypic control. Neutrophils were identified by their forward/scatter characteristics within the CD45^+^ population, comprising >99% CD66b^+^ and <1% CD14^+^ events. The normal range of the neutrophil MPO content was defined on the basis of the results obtained in 50 healthy subjects. In order to establish the background level of MPO-associated fluorescence after complete MPO depletion, the neutrophil MPO content was also assessed after stimulation of whole blood samples with fMLP (0.5 µM) in the presence of cytochalasin B (2.5 µg/mL) to induce the full release of neutrophil azurophilic granules content [28]. We used the intermediate range of MPO-associated MFI to define neutrophils with low MPO content. (**Figure S1**). Platelet leukocyte heterotypic aggregates, platelet P-selectin, leukocyte ß2 upregulation and transactivation as well as leukocyte MRP8/14 expression were measured as previously described [9], [10], [11], [26]. Analysis of samples was with a FC500, Beckman-Coulter.

**Figure 2:**
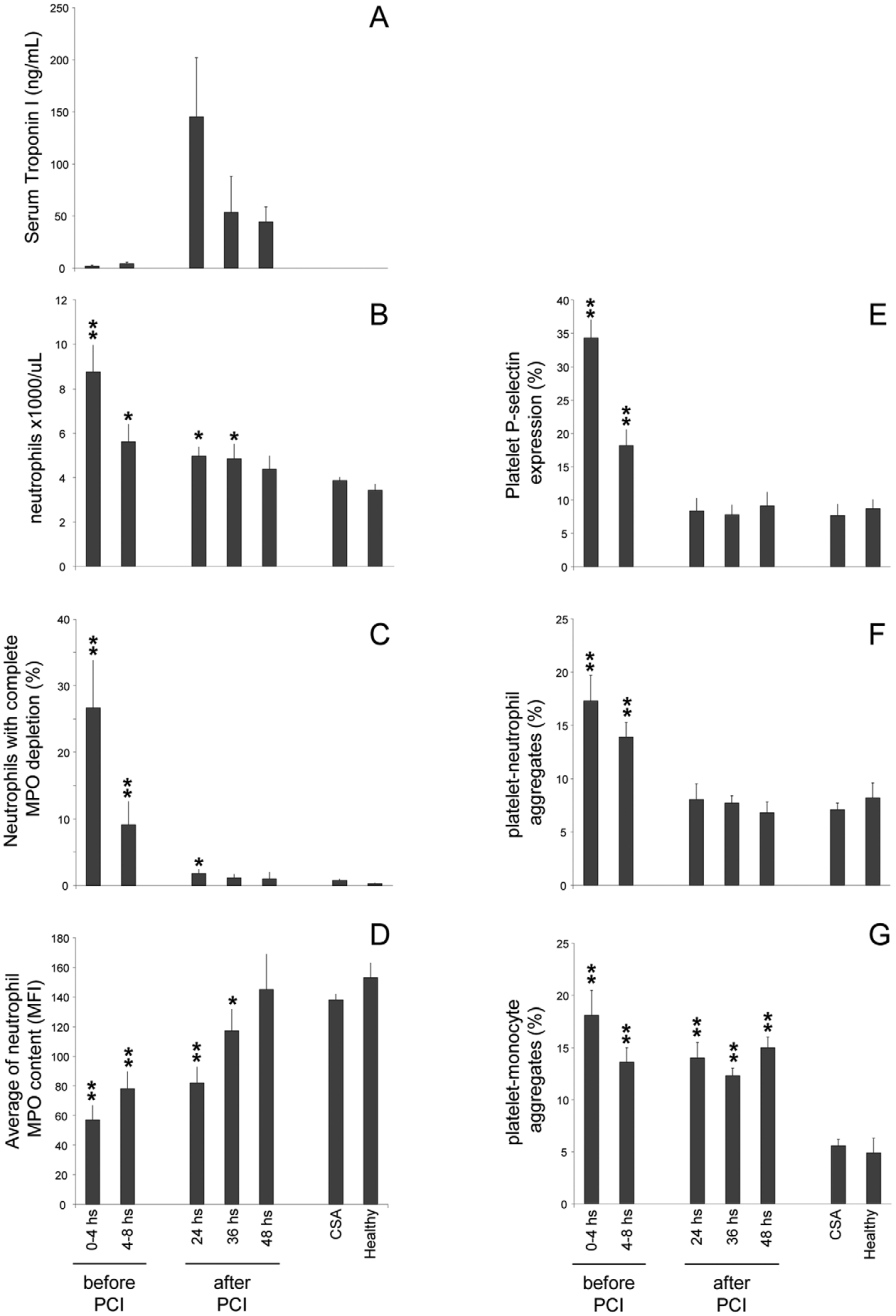
Time course of leukocyte and platelet activation in patients and controls. 36 patients with acute myocardial infarction were studied before any therapeutic treatment and re-studied at different times on PCI. Results (expressed as mean ± SEM) were compared with 69 patients with chronic stable angina candidates (CSA) for PCI and 138 healthy donors. Determinations were performed by flow cytometry, with the exception of troponin I, as described in material and methods. Y axis: serum troponin (Panel A), granulocyte cell counts (panel B), fraction of circulating neutrophils with complete myeloperoxidase (MPO) depletion (Panel C), average of neutrophil MPO content (Panel D), fraction of platelet expressing P-selectin (Panel E), the fraction of platelet-neutrophils heterotypic aggregates (Panel F), fraction of platelet-monocyte heterotypic aggregates (Panel G). * = *P*<0.05; ** = *P*<0.01 (both respect to CSA and healthy donors) were determined by ANOVA followed Bonferroni test.

### Animal Model

Whole blood from female C57BL/6N mice (wild type) or from C57BL/6-P-selectin (P-sel ^−/−^) was obtained, processed and activated as described [10], [26]. Platelets were injected in the tail vein of wild type mice. After 1, 2, 3, and 24 hours blood was obtained from the tail. The neutrophil MPO content and platelet-neutrophil heterotypic were determined by flow cytometry as described [10], [26].

**Figure 3:**
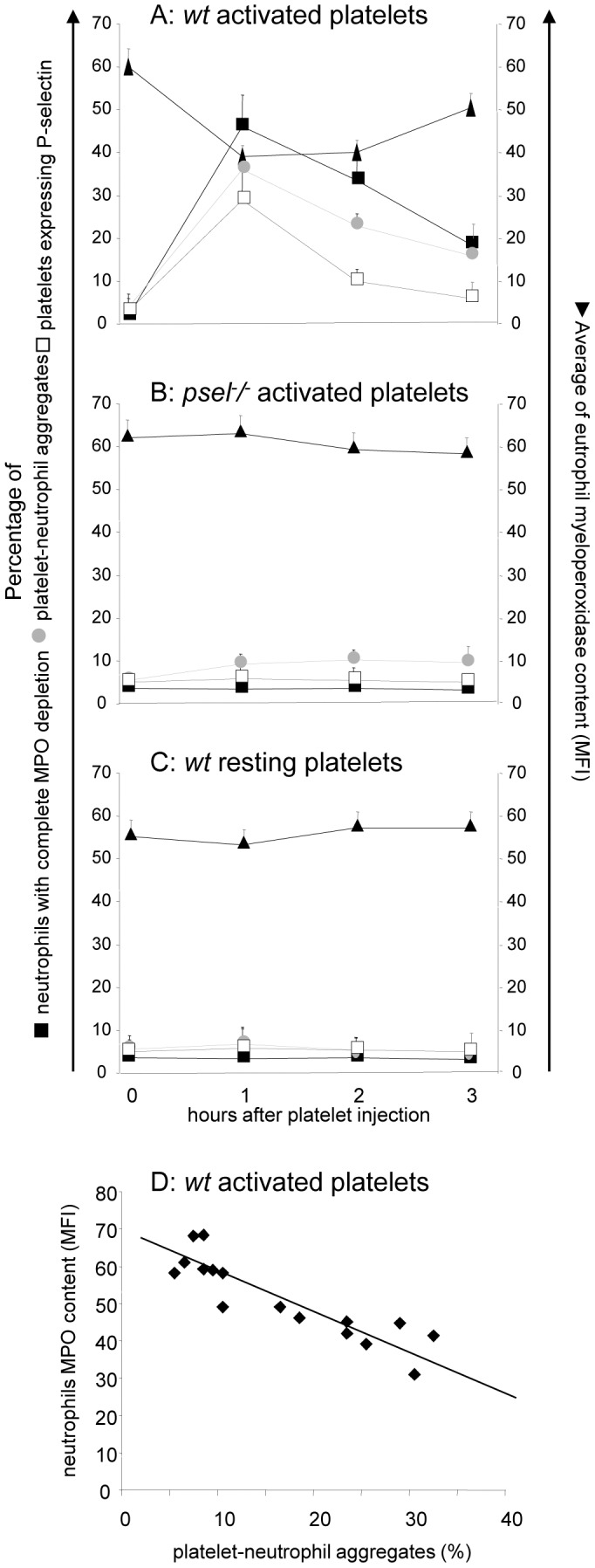
In vivo evidence that circulating activated platelets cause neutrophil degranulation. Purified platelets from wild type or Psel ^−/−^ mice were activated (or not) and injected in the tail vein of wild type C57BL/6 mice. Platelet activation was achieved with thrombin. The molecule was then inactivated by addition of hyrudin. Neutrophil myeloperoxidase content, platelet P-selectin expression and platelet-neutrophil heterotypic aggregates were assessed by flow cytometry as described in the Methods. Panel A shows the effect of the in vivo injection of the buffer used for platelet activation, containing both thrombin and hyrudin. Blood was retrieved at different times after injection (hours, x axis) and neutrophils with adherent (filled symbols) or internalized (grey symbols) platelets were identified by flow cytometry. >70% of circulating neutrophils were engaged in the internalization and adhesion of activated, but not resting, platelets after 1 hour. The fraction of circulating neutrophils with internalized and adherent platelets returned to background levels 24 hours after the injection. Results are expressed as mean±S.E.M. 5–7 animals were assessed per each point.

## Results

### Circulating Neutrophil Degranulation

The neutrophil MPO content was assessed by flow cytometry and by confocal microscopy in whole blood samples fixed and labeled without any further manipulation. The normal range of neutrophil MPO content, preliminarily defined in our laboratory on the basis of the results obtained on 50 healthy subjects, ranged between 98 and 203 arbitrary units of mean fluorescence intensity (MFI) detected within the neutrophil (CD66b^+^) population. Background signal (after complete neutrophil MPO depletion by stimulation of whole blood samples with fMLP) was to 8–22 MFI. Thus the 23–97 MFI range was used to define neutrophils with low MPO content (**Figure S1**).

The average neutrophil MPO content in healthy donors (149.3±3.4 MFI) was stable: it did not substantially change with age (age range 20–80 years) or gender (**Table S1).** The MPO content in contrast was dramatically reduced in patients with ACS (**Table 4**, **Figure 1** and **Figure S1**), but it was unaffected, either before or after PCI, in patients with CSA who had a greater burden of coronary atherosclerosis than ACS patients (**Table 1** and **Table 4**).

Neutrophils from STEMI patients had a significantly greater reduction of the average MPO content compared to NSTEMI patients and patients with unstable angina (56.1±5.8 *vs* 73.2±10.4 and *vs* 91.7±12.0 MFI, p<0.005). Interestingly, a trimodal distribution of neutrophil MPO was found in all but one STEMI patients: complete depletion in 21.5±5.4% of neutrophils (7.5±1 MFI), reduced content in 46.5±7% of neutrophils (51.3±3.7 MFI), and normal content in 30.6±7.1% of neutrophils (139.6±14.4 MFI) (**Figure 1** **,** panels **A-D,** **Table 4** **).**

The simultaneous occurrence of three different sub-populations of neutrophils was confirmed by confocal microscopy (**Figure 1** **,** panels **B**). Nine of 21 NSTEMI patients also had a trimodal pattern of MPO expression. By contrast, neutrophils from patients with unstable angina had a homogeneously reduced unimodal expression of MPO (**Table 4**). Neutrophil degranulation was significantly associated with up-regulation and transactivation of the leukocyte ß2 integrin (CD11b/CD18) Mac-1 (**Table 5**).

CD16 expression is reduced or absent in apoptotic neutrophils ([29], [30], [31]).The possibility that complete MPO depletion observed in AMI patients was due to neutrophil apoptosis was ruled out by the observation that 94.1±1.8% of neutrophils with complete MPO depletion in 15 consecutive AMI patients had a normal expression of CD16 (859.1±22.2 MFI) compared to that of healthy matched controls (96.6±1.3%, 855.2±15.4 MFI). At variance, the subpopulation of neutrophils with normal MPO content was positive for MRP8.14, possibly reflecting its recent release into the bloodstream (**Table 5**).

### Time Course of MPO Depletion

The percentage of neutrophils with a complete MPO depletion was greatest in STEMI patients studied within 4 hours, intermediate in those studied within 4–8 hours and lower in samples collected 8–12 hours after the onset of symptoms (in all cases prior to any treatment) (**Figure 2**). The average neutrophil MPO content returned to normal values within 24–36 hours (**Figure 2**).

Neutrophil activation was associated with monocyte activation, as indicated by CD11b/CD18 up-regulation and transactivation (**Table 5**). In all ACS patients a significant increase of platelet-leukocyte (neutrophils and monocytes) heterotypic aggregates was observed (**Table 5** **,** **Figure 1** **,** panels **E-F** and **Figure 2**). This was maximal for neutrophils in samples taken early after the onset of symptoms and disappeared within 24 hours (**Figure 2** **,** panel **F**). Monocyte activation persisted after 48 hour, possibly because of monocyte longer life span (**Figure 2** **,** panel **G**). Circulating platelets were also activated, as indicated by the up-regulation of surface P-selectin (**Table 5**). The time course of platelet activation was similar to that of neutrophils (**Figure 2** **,** panel **E**).

### Neutrophil Activation in Systemic Inflammatory Conditions

To investigate whether the trimodal pattern of MPO expression observed in the very early phases of STEMI was also present in other systemic inflammatory syndromes, we studied conditions of leukocyte activation associated with microbial infection (sepsis) or with self-sustained systemic inflammation (rheumatoid arthritis, ANCA-associated small-vessel vasculitis, polymyalgia rheumatica and giant cell arteritis) (**Tables 2** **and** **3**). A reduced neutrophil MPO content was observed in all these clinical conditions. The trimodal pattern of MPO expression observed in AMI patients was not detectable in any of the other conditions, regardless of: *i*) increased peripheral neutrophil count in patients with acute traumatic bone fracture (12.8±0.3 neutrophils/µL) and sepsis (22.2±4.4 neutrophils/µL); *ii*) the presence of ANCA, known to amplify the activation of neutrophils [12], and *iii*) the massive elevation in inflammatory markers of patients with giant cell arteritis, polymyalgia rheumatica or rheumatoid arthritis **(** **Table 4**). A normal MPO content was observed in the four patients undergoing septal alcoholization; taken together with the normal MPO content observed in patients with bone fracture these data strongly suggest that tissue necrosis *per se* is not responsible for the trimodal pattern of MPO expression observed in AMI patients **(** **Table 4**).

### Neutrophils MPO Depletion and Platelet Activation

As platelet activation has been associated to neutrophil degranulation [10], [11], [28] we have also assessed platelet expression of P-selectin and the presence of platelet-neutrophil heterotypic aggregates in STEMI and NSTEMI patients (**Figure 1** **,** panel **G** and **Table 5**). At covariance analysis after logarithmic transformation of data the percentage of platelets expressing P-selectin was an independent variable (p = 0.00016) for platelet-neutrophil heterotypic aggregates and neutrophil MPO content. Furthermore, the percentage of platelets expressing P-selectin was inversely correlated with the average neutrophil MPO content (r = −0.75, p<0.001) and directly correlated with the percentage of neutrophils with complete MPO depletion (r = 0.57, p<0.009) in AMI patients (**Figure 1** **,** panel **G)**. Accordingly, the fraction of platelet-neutrophil heterotypic aggregates correlated inversely with the average MPO content (r = −0.53, p<0.0005) and directly with the proportion of neutrophils with complete MPO depletion (r = 0.41, p<0.001).

Platelet and neutrophil activation were associated, because the extent of platelets adhesion (heterotypic aggregates) was significantly higher for neutrophils with complete MPO depletion than with low MPO content. Panels **E-F** of **Figure 1** depict the analysis of patients samples by confocal microscopy and flow cytometry simultaneously: only very few adherent platelets were observed in neutrophils with normal MPO content.

Patients with rheumatoid arthritis and giant cell arteritis showed a variable proportion of neutrophils with adherent platelets, with an inverse correlation between the percentage of platelet-neutrophil heterotypic aggregates and the neutrophil MPO content (r = 0.59, P<0.01). No correlation was found in patients with ANCA- associated small vessel vasculitis or with severe sepsis.

### Platelet P-selectin Expression is Required for MPO Depletion in Experimental Animals

We have injected purified platelets, either activated or resting, in the tail vein of C57BL/6 mice. Blood was sampled at 1, 2, 3 and 24 hours after platelet infusion and the neutrophil MPO content was determined by flow cytometry. One hour after the injection of activated platelets the percentage of platelets expressing P-selectin increased from 2.2±1.3% to 29.0±1.1% (p<0.001) and 46.0±5.5% of circulating neutrophils showed complete MPO depletion (**Figure 3**). The fraction of platelet-neutrophil heterotypic aggregates increased from 5.7±2.1 to 36.2±2.3% (p<0.001) while the neutrophil MPO content decreased from 58.2±1,6 to 38.9±1.0 (p<0.001); **Figure 3** **, panel A**). No significant change in any of these parameters was observed in mice injected with activated platelets from P-sel^−/−^ mice (**Figure 3** **, panel B**), or with resting platelets (**Figure 3** **, panel C**).

## Discussion

Our results indicate that neutrophil activation is substantially greater in patients with STEMI, compared with those with non STEMI or unstable angina. Neutrophil activation is associated with increased neutrophil counts and with a larger fraction of circulating neutrophils with features reflecting recent origin from the bone marrow. Of importance, a fraction of neutrophils is not degranulated in STEMI patients, which may reflect an abnormal response to activating stimuli or, more likely, a population of circulating neutrophils never exposed to stimuli restricted within a specific vascular district. The acute arterial occlusion that is an hallmark of myocardial infarction could contribute in these patients to protect neutrophils newly emerged from the bone marrow, preventing MPO release.

Neutrophil activation is strictly linked to systemic inflammation, because microbial and endogenous triggers of inflammation are primarily recognized by innate receptors which are non clonally distributed on circulating leukocytes. Not surprisingly, a normal MPO content was observed in only 2% of neutrophils from patients with severe sepsis. Mobilization of the neutrophil effector action, and specifically the release of the granule content, could in principle jeopardize the integrity of the vessel wall [2], [3], [20], [32], [33], and several regulatory feedback mechanisms regulate the extent of neutrophil activation in the peripheral blood [34]. Accordingly, we failed to detect the trimodal pattern of MPO expression in any of the self-sustaining inflammatory conditions investigated, where the original *noxa* is likely to be persistent, but possibly expressed at a lower extent compared to STEMI.

A low neutrophil MPO content was found in patients presenting with very low levels of troponin I, a well-accepted marker of myocardial cell death (**Table 1**, **Figure 2A**), suggesting that neutrophil activation is an early event in patients with evolving AMI. From a pathophysiological perspective, it is intriguing to speculate whether neutrophil activation may even precede myocardial injury and may be causally involved in plaque rupture [2], [3], [20]. It cannot be excluded that the systemic release of markers of tissue injury occurs at a later time compared to MPO release. However, the marked differences in time course raise the possibility that neutrophil activation associated to myocardial necrosis may represent a consequence or even a component of the evolving plaque rupture. Other studies speculated on this tenet and neutrophil degranulation and the consensual increase of neutrophil-derived proteases in plasma were found in patients with unstable angina and acute myocardial infarction.

Tissue necrosis itself could incite neutrophil activation [6] [35]. To address this issue, we have verified the effect of percutaneous transluminal septal myocardial ablation, a procedure based on the injection of alcohol into the septal branches of the coronary artery that feed the enlarged interventricular septum of patients with hyperthrophic obstructive cardiomyopathy. This procedure results in the synchronized death of myocardiocytes, with substantial and swift increase of troponin I blood levels and represents a unique human model of iatrogenic myocardial necrosis *in vivo* [24], [36]. We did not detect any MPO content reduction. The result, if combined with the conserved MPO content of neutrophils after PCI or acute traumatic fracture of the bone, makes unlikely the possibility that necrosis *per se* is sufficient to account for MPO depletion. Of interest, in none of these circumstances we have observed increased levels P-selectin or of P-selectin dependent events, such as heterotypic platelet-leukocyte aggregates.

Moreover, patients with CSA had substantial atherosclerosis of coronary arteries, but circulating neutrophils had a relatively normal MPO content. This observation may suggest that atherosclerosis *per se* is not directly involved in MPO depletion, which appears to be specifically associated with acute coronary syndromes.

The persistence of high plasmatic levels of MPO can identify patients with high risk of complications [32], [37]. This study to our knowledge is the first directly comparing the time courses of leukocyte activation and myocardial tissue damage. This is of importance because systemic leukocyte activation is widely believed to occur as a consequence of myocardial tissue injury rather than of plaque instability preceding the myocardial infarction. Our data now indicate that this perception might underestimate the cross talk between neutrophils and activated platelets in acute coronary syndromes and well fit with the previous observations of the widespread activation of neutrophils across the coronary vascular bed [38].

We have observed a tight correlation between indices of platelet-neutrophil interaction: the characteristic feature of MPO content, together with the results obtained in the experimental animal model, suggest that neutrophil degranulation is due to their interaction with activated platelets. Indeed, previous experimental models support the concept that P-selectin is able to induce neutrophil activation and degranulation [9], [10], [11], [28], [39]. The time course of P-selectin expression observed by us and others [1], [10], [11], [17] and the evidence that platelet P-selectin is able *in vitro* [10], [11], [28] and *in vivo* to prompt the degranulation of neutrophil are in agreement with this model. Our experimental evidences indicate that a burst of P-selectin expression (such as that induced in vivo by the injection of activated wild-type but not P-selectin knock-out platelets) reproduces the extreme and transient neutrophil MPO depletion that we have observed in patients with acute myocardial infarction. These observations may have diagnostic and pathogenetic implications.

Neutrophil MPO complete depletion occurs when platelets express P-selectin rather abruptly, like it occurs in patients with AMI, in which a sudden and transient burst of platelet activation occurs. This is the scenario that we mimic in the mouse, in which we directly inject in the blood P-selectin expressing platelets. In contrast, platelets of patients with unstable angina or with other systemic inflammatory syndromes are characterized by a persistent, waxing and waning P-selectin expression. This is likely to recruit adaptive mechanisms that limit the paroxysmal neutrophil activation and thus protect the integrity of vessel walls. Further studies are necessary to verify this possibility.

The transient nature of neutrophil activation in patients with AMI could be explained by rapid loss of P-selectin as a consequence of active proteolysis [1], [39] by the proteases derived from the neutrophils and by the inhibitory effect of pentraxin 3 released by neutrophils as consequence of activated platelet adhesion [8], [11], [40], which has been shown to interfere with neutrophil functions in vitro and in vivo [41]. Moreover, the clearance of activated platelets represents a further likely mechanism involved in the swift termination of neutrophil activation [7], [8], [10], [26]. The actual contribution of these non-mutually exclusive mechanisms remains to be established.

## Supporting Information

Figure S1 **Intracellular MPO content in resting and stimulated neutrophils assessed by confocal microscopy and flow cytometry.** Whole blood samples from healthy donors were stimulated as described in material and methods. For confocal determinations, monoclonal antibodies against MPO were labeled with Alexa Fluor 488 (green) and DNA labeled with Hoechst (blue). For flow cytometry, samples were labeled with mAbs against CD66b (specific for neutrophils) and after permeabilization with mAbs against MPO.
(TIF)

Table S1 **MFI =  mean fluorescence intensity.**
(DOC)
