# Supplementary figures and images for: An Intense and Short-Lasting Burst of Neutrophil Activation Differentiates Early Acute Myocardial Infarction from Systemic Inflammatory Syndromes
Source: PLoS One. 2012 Jun 25;7(6):e39484. doi: 10.1371/journal.pone.0039484 (PMC3382567; doi:10.1371/journal.pone.0039484)

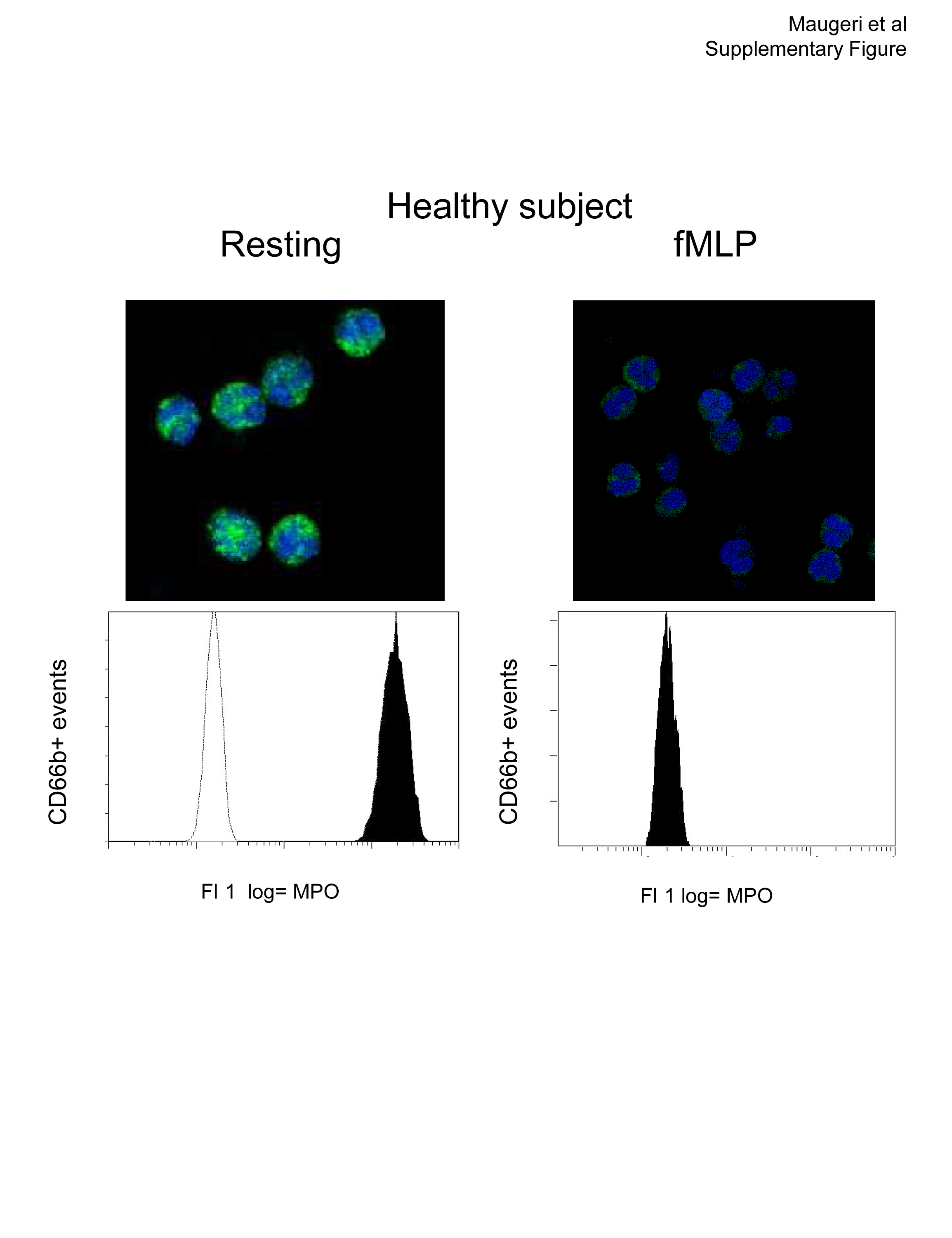

Supplement: Figure S1 — Intracellular MPO content in resting and stimulated neutrophils assessed by confocal microscopy and flow cytometry. Whole blood samples from healthy donors were stimulated as described in material and methods. For confocal determinations, monoclonal antibodies against MPO were labeled with Alexa Fluor 488 (green) and DNA labeled with Hoechst (blue). For flow cytometry, samples were labeled with mAbs against CD66b (specific for neutrophils) and after permeabilization with mAbs against MPO. (TIF) [file pone.0039484.s001.tif]
