# Supplementary material for: An Intense and Short-Lasting Burst of Neutrophil Activation Differentiates Early Acute Myocardial Infarction from Systemic Inflammatory Syndromes
Source: PLoS One. 2012 Jun 25;7(6):e39484. doi: 10.1371/journal.pone.0039484 (PMC3382567; doi:10.1371/journal.pone.0039484)
Supplement: Table S1 — MFI = mean fluorescence intensity. (DOC) [file pone.0039484.s002.doc]

**Table S1**

Neutrophil myeloperoxidase (MPO) content in healthy donors is independent of age and gender.

| Age (years) | n | Neutrophils count  (x10^3^/µL) | Average MPO  content  (MFI) | MPO  depletion  (% neutrophils) | Low  MPO content  (% neutrophils) | Normal  MPO content  (% neutrophils) | Trimodal MPO content  (N°/total) |  |
| --- | --- | --- | --- | --- | --- | --- | --- | --- |
| 20-29 | 30 | 3.6±0.2 | 150.5±6.4 | <1 | 2.2±0.7 | 93.1±5.0 | 0/30 | |
| 30-39 | 34 | 3.7±0.2 | 148.4±5.2 | <1 | 0.5±0.3 | 97.7±0.4 | 0/34 | |
| 40-49 | 30 | 3.6±0.1 | 147.2±9.0 | <1 | 0.5±0.2 | 96.4±4.5 | 0/30 | |
| 50-59 | 18 | 3.5±0.3 | 151.5±9.2 | <1 | 0.6±0.3 | 95.6±4.3 | 0/18 | |
| 60-69 | 16 | 3.6±0.5 | 149.4±9.6 | <1 | 0.3±0.2 | 95.1±3.7 | 0/16 | |
| 70-80 | 10 | 3.1±0.3 | 145.5±10.8 | <1 | 1.3±1.0 | 93.1±1.2 | 0/10 | |
| Male | 74 | 3.5±0.8 | 150.9±31.1 | <1 | 2.2±1.0 | 92.0±4.1 | 0/74 | |
| Female | 64 | 3.6±0.5 | 146.0±38.5 | <1 | 1.3±0.8 | 95.1±8.0 | 0/64 | |
